# ALSPAC Mercury Study and Fish Consumers: Golding et al. Respond

**DOI:** 10.1289/ehp.1307757R

**Published:** 2014-02-01

**Authors:** Jean Golding, Colin D. Steer, Tony Lowery, Joseph R. Hibbeln

**Affiliations:** 1 Centre for Child and Adolescent Health, University of Bristol, Bristol, United Kingdom; 2 National Seafood Inspection Laboratory, National Marine Fisheries Service, National Oceanic and Atmospheric Administration, Pascagoula, Mississippi, USA; 3 National Institute on Alcohol Abuse and Alcoholism, National Institutes of Health, Department of Health and Human Services, Bethesda, Maryland, USA

We apologize if our article (Golding et al. 2013) was unclear in some respects. We would like to respond to the comments of Gochfeld et al. and clarify some points in our article.

Contrary to the claims by Gochfeld et al., the Avon Longitudinal Study of Parents and Children (ALSPAC) study was designed to investigate the effects of various prenatal measures of the environment (including analyzing blood samples for trace metals and estimating dietary intake); the initial aim was to determine the relationship of these measures to a variety of different outcomes.

Gochfeld et al. state that “< 2% of the ALSPAC subsample” were women who ate fish frequently (> 3 portions a week). However, when consumers of both oily and white fish are combined, the frequent fish-eaters actually comprised 647 (18%) of our pregnant population.

In our article (Golding et al. 2013), we reported that “blood levels exceeded the 5.8 μg/L reference dose level … in 38 women (0.92%).” A reanalysis of our data in regard to the 647 frequent fish consumers showed that only 2.0% of them had high mercury levels (> 5.8 µg/L).

In their letter, Gochfeld et al. cite two references (Björnberg et al. 2005; Mahaffey et al. 2004) to support their statement that “for most people, fish is the only significant source of methylmercury”—but neither of these studies assessed the contributions of mercury from any specific dietary source other than fish.

The categorical nature of our food frequency questionnaire successfully characterizes fish consumption in the study population; people do not eat the same type of fish regularly but typically consume different types of seafood. Internal validation of our fish exposure estimates shows that they are correlated with biological markers of fish consumption, such as omega-3 fatty acids (Williams et al. 2001). The measures we used reflected typical distributions of consumption of commercially available fish in the area. It should be noted that our results are based on population averages. Finer gradations in descriptions of fish consumption are not likely to substantially alter the results.

In response to the statement of Gochfeld et al. that our study “did not address the risk for the frequent fish-eater who has elevated blood mercury,” we analyzed separately the group of frequent fish consumers (> 3 portions/week). We found that members of this group had a similar or lower contribution of blood mercury from seafood than the less-frequent consumers.

Gochfeld et al. suggest that published benefits of fish consumption may generally be a result of healthy lifestyles in general. In a previous study (Hibbeln et al. 2007), we performed a sensitivity analysis using paternal fish consumption. We found little association between offspring IQ (intelligence quotient) and paternal fish consumption compared with maternal fish consumption, implying that the maternal fish consumption effect was unlikely to be caused by social patterning.

As we note in our paper (Golding et al. 2013), detailed analyses of typical British diets have shown that fish provide between 25% (Ysart et al. 1999) and 33% (Ysart et al. 2000) of total dietary mercury. Thus, our figures are not as surprising as might have been expected.

We were surprised that Gochfeld et al. did not highlight the important finding of contributions to mercury levels from foods that have no obvious nutrient value, such as herbal drinks, which we found to have an important association with blood mercury levels. Warning against such drinks may have greater net benefits to pregnant women than reduced fish consumption.

Finally, we agree with Gochfeld et al. that pregnant women might benefit slightly by substituting fish species containing very high methylmercury with those having lower levels, but we do not recommend that women cut down their overall fish consumption.
